# Binswanger's disease: Case presentation and differential diagnosis

**DOI:** 10.1002/ccr3.3459

**Published:** 2020-10-27

**Authors:** Vitalie Văcăraș, Adrian Mihai Cordoș, Imelda Rahovan, Sorina Frunze, Dafin Fior Mureșanu

**Affiliations:** ^1^ Iuliu Haţieganu University of Medicine and Pharmacy Cluj‐Napoca Cluj‐Napoca Romania; ^2^ Neurology Department Cluj‐Napoca County Emergency Hospital Cluj‐Napoca Romania

**Keywords:** acute medicine, cardiovascular disorders, neurology, psychiatry

## Abstract

Establishing a diagnosis of Binswanger's disease requires a multimodal approach. As new pathophysiological mechanisms are revealed, tests that should yield greater specificity will become available in the years to come.

## Graphical abstract

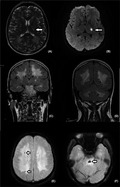

## 1 INTRODUCTION

The term “leukoencephalopathy” refers to a heterogeneous group of disorders characterized by the degeneration of the white matter of several etiologies: vascular, toxic, infectious, and genetic. The last group includes the so‐called leukodystrophies.^1^

The term Binswanger's disease was given by Alois Alzheimer in 1902 in honor of his professor, Otto Binswanger, who first described the clinical and pathological aspects of the disease in 1884.^2^ Binswanger's disease, or “subcortical arteriosclerotic encephalopathy,” as Olszewski called it 60 years after its first discovery,^3^ refers to a type of leukoencephalopathy linked to circulatory and vascular factors with significant clinical consequences frequently associated with arterial hypertension, arteriosclerosis, and strokes.^4^

Binswanger's disease represents one of the causes which lead to vascular cognitive impairment alongside cerebral lacunes, amyloid angiopathy, and some forms of Alzheimer diseases, and it may coexist with any of these disorders.^5^

While vascular dementia is generally considered the second most common subtype of dementia, after Alzheimer disease, accounting for roughly 15%‐20% of dementia cases in North America and Europe,^6^ the epidemiology of Binswanger's disease is still not well studied.^5^

Louis Caplan established in 1995 the first criteria for that are required for diagnosis, and they are subdivided into three categories which we will enunciate briefly in the following paragraphs.^2^ These criteria still hold to the present day and have been adapted through the course of time along with a better understanding of the physiopathological and morphopathological characteristics.

### 1.1 The presence of known or hypothesized risk factors

The most important and frequently described risk factor associated with Binswanger disease is chronic, uncontrolled, arterial hypertension; therefore, its absence in a patient with cognitive impairment and neurological signs should lead to questioning the diagnosis.^5^ The explanation most often proposed is that chronic arterial hypertension is responsible for the narrowing of the small blood vessels due to lipohyalinosis and fibrosis with subsequent blood flow reduction and hypoxia. These phenomena lead to a local neuroinflammatory response which in turn results in myelin sheath degeneration.^7^ Other risk factors such as diabetes mellitus, smoking, dyslipidemia, sleep apnea, and atrial fibrillation, although frequently present in these patients, have a smaller role in establishing the diagnosis of the disease.^5^ Exclusion of other diseases which lead to white mater degeneration, such as multiple sclerosis, AIDS, or radiation toxicity is crucial.^2^

### 1.2 Clinical features

An essential element lies in the way the clinical aspects of the disease evolve, with stepwise or gradual progression of the cognitive impairment and other neurological signs and symptoms.^2^, ^5^, ^8^ First symptoms usually appear between the fifth and the seventh age decade.^8^ The clinical course of the illness is variable and evolves over a 5‐ to 10‐year period. There does not seem to be any gender bias. Cognitive and behavioral changes are characterized by dementia and a dysexecutive syndrome (changes in attentional control, working memory, and short‐term memory, impulse control, and abulia in the final stages).^2^, ^5^ Computations and mathematical functions are usually deficient.^2^ Abnormalities of long‐term memory, language, and visual‐spatial functions are not as prominent as in patients with Alzheimer's or Pick's disease and therefore the MMSE (Mini Mental State Examination) can often be within normal range, while the MOCA score (Montreal Cognitive Assessment) may evidence cognitive impairment.^5^ History often reveals past strokes which can be sometimes typical to one of the multiple lacunar syndromes, pure motor hemiparesis being the most frequent of them.^8^ In other patients, the focal neurologic deficits can have a subacute onset with progressions during days or weeks and are sometimes associated with strokes. Cognitive and behavioral impairment, motor and gait disturbances, falls, and incontinence evolve with periods of stabilization, plateaus, and periods of improvement. A mixture of pyramidal tract signs, extrapyramidal signs, and pseudobulbar signs can often be seen.^2^, ^5^, ^8^

### 1.3 Imaging

The first imaging descriptions of the lesions were given using computed tomography (CT). The ubiquitous characteristic of the illness is represented by the changes to the subcortical white matter which has a bilateral presentation, described as low dense lesions without contrast enhancement. These lesions are most often present in the periventricular regions, especially adjacent to the frontal horns. These changes were named leukoaraiosis by Hachinski and they denote the rarefaction of the subcortical white matter. Juxtacortical white matter (“U”‐association fibers) is often spared. It is important to mention that these changes can be present without any neurological signs and can be also associated with aging.^8^ White matter disorders are better characterized on MRI which has a greater sensitivity than CT. The areas of demyelination are described on MRI as large, confluent, white mater hyperintensities (on T2WI and FLAIR sequences), with ill‐defined borders. The lesions are discretely hypointense on T1WI sequences. Lesions are usually bilateral, symmetric, and grouped around the frontal horns, but can have variable degrees of extension, and both the periventricular and deep white matter can be affected, but the juxtacortical white matter is always spared, as mentioned before. Subcortical lacunes and “mini‐strokes” are often found and the Virchow‐Robin perivascular spaces are frequently enlarged.^4^, ^5^, ^9^ Lesions can also be present in the white matter of the brain stem, especially the central pons (the medulla oblongata and the midbrain are also more often than not spared).^10^ Mild to moderate white matter atrophy is also a common finding.^5^ Diffusion‐weighted imaging (DWI) can detect acute ischemic lesions. Subcortical microbleeds can be seen in Binswanger's disease and can be detected on SWI sequences, but their presence in large numbers or if they are located in the cortical regions should raise the suspicion of amyloid angiopathy.^5^

## 2 CASE PRESENTATION

A 50‐year‐old Caucasian man, residing in an urban area, with right laterality and no history of any chronic illnesses, was admitted to our Neurology department with the complaint of weakness in the right limbs. The patient's symptoms had an acute onset 2 days prior to presentation. Family history revealed that the patient's mother suffered from an ischemic stroke at the age of 87. The patient was an artist and a painter, admitted to being a cigarette smoker (1 pack of cigarettes per day for over 30 years) and to consuming alcohol daily in the last 10 years (about 50 cmc of spirits per day equivalent to 2 units of alcohol per day). There was no history of head trauma or any known allergies.

The general examination revealed that the patient was conscious and aware and had normal body temperature, no signs of recent trauma, and a BMI (Body Mass Index) of 21 kg/m^2^. The blood pressure was 234/146 mm Hg, and the heart rate 104 beats per minute.

The neurological examination showed the following:

1. Pyramidal tract signs characterized by hemiparesis regarding the right limbs with a score of 4/5 (on the MRC—Modified Research Council scale). Extensor plantar reflex was objectified in the right leg. The patient also had central face palsy on the same side.
2. Extrapyramidal signs characterized by slowness, left upper limb rigidity, hypomimia and a low‐volume, monotonous speech.
3. Mild cognitive impairment on MMSE testing (a score of 27/30) and on MOCA testing (25/30). The abilities affected in our patient were visuospatial/executive functions, short‐term memory, and mathematical functions.

### 2.1 Paraclinical investigations

**FIGURE 1:**
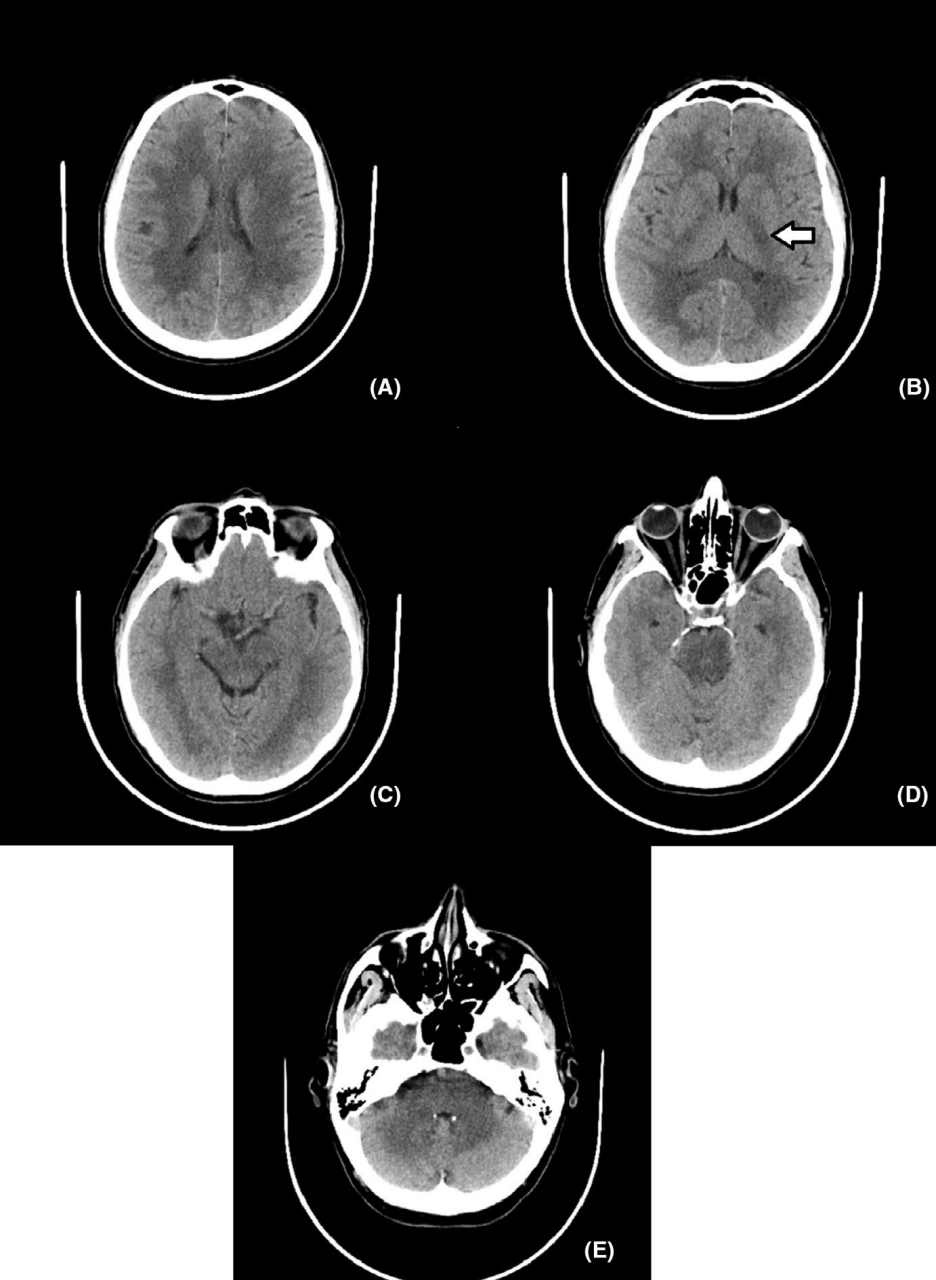
Computed tomography images showing diffuse white matter hypodensities in the centrum semiovale (A), inner capsule (B), midbrain (C), pons (D), and cerebellum and medulla oblongata (E). The arrow shows a small hypodense lesion in the posterior limb of the left internal capsule

On admission a head CT without contrast dye was performed (Figure 1A‐E). The CT revealed a small hypodense lesion (Figure 1B), with ill‐defined borders, located in the posterior limb of the left inner capsule that was interpreted as an acute lacunar stroke.

Diffuse white matter hypodensity was observed, with symmetrical pattern, regarding the periventricular region, the centrum semiovale, and the inner capsule. The juxtacortical arcuate fibers were spared. The hypodense white matter lesions extended in the brainstem, cerebellar peduncles, and the cerebellum.

**FIGURE 2:**
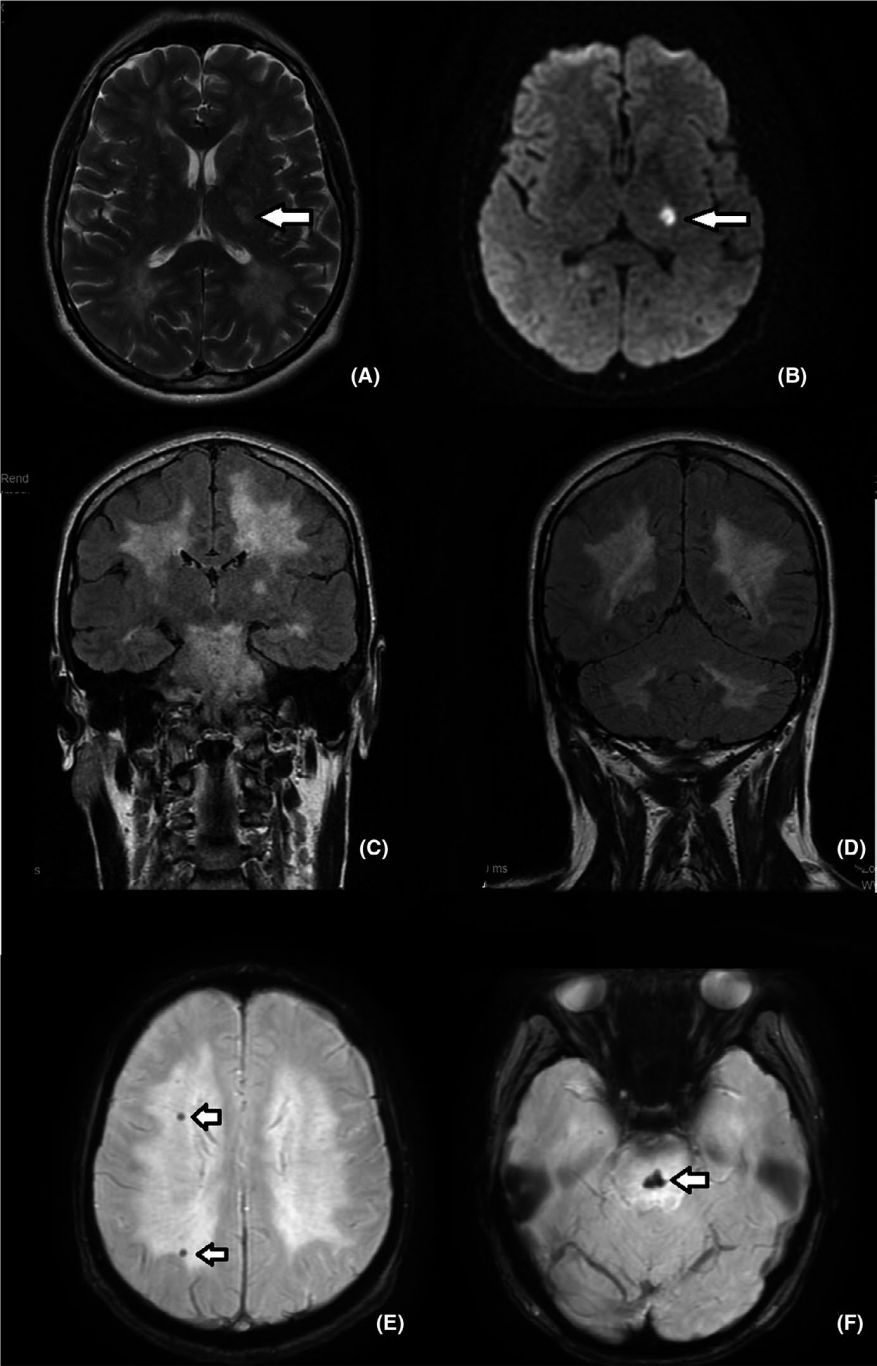
A, Axial cross‐section MRI image of the brain, T2 weighted, revealing diffuse white matter hyperintensities. B, DWI sequence. Arrows indicate to a hyperintense lesion located in the posterior limb of the left internal capsule (A, B). C, D, Coronal cross‐section MRI images on FLAIR sequence, revealing supratentorial and infratentorial white matter hyperintensities. E, F, SWI sequence. Arrows indicate to hemosiderin deposits in the centrum semiovale (E) and the pons (F)

For a more precise evaluation of the white matter changes, a native MRI was performed (Figure 2A‐F).

MRI revealed an acute ischemic lesion (10/19 mm), hyperintense on the DWI, FLAIR, and T2WI sequences (Figure 2A,B), located in the posterior limb of the inner capsule that extended to the lenticular nucleus on the left. Diffuse hyperintense areas on T2WI and FLAIR sequences were also observed in the white matter, with the same distribution (supratentorial and infratentorial) as the hypodense areas described on head CT (Figure 2A‐D), without abnormal restricted diffusion (Figure 2B). On the SWI sequences, conglomerate hypointense lesions adjacent to the midline of the pons ware observed (Figure 2F) that were interpreted as old hemosiderin deposits. Similar lesions were observed in the centrum semiovale (Figure 2E).

Complete bloodwork was done. No notable laboratory changes regarding the hematological profile, liver and kidney functions, and coagulation parameters were observed. No biological inflammatory syndrome was present on admission and during hospitalization. Other notable bloodwork parameters will be mentioned below in the differential diagnosis subchapter. ECG was within normal range.

A carotid Doppler ultrasonography was performed which showed bilateral nonstenotic atheromatous plaques, with heterogenous echogenicity, irregular surface, and a thickness ranging from 2.7 mm in the left common carotid artery to 4.2 mm in the right common carotid artery were observed.

A positive diagnosis of Binswanger's disease, lacunar stroke in posterior limb of the left inner capsule associated with pure motor hemiparesis regarding the right side of the body, and stage III arterial hypertension at presentation was established.

#### 2.1.1 Differential diagnosis

Using the imaging aspects of the disease on MRI, we decided taking into account for the differential diagnosis disorders which are compatible with the pattern of the white matter lesions as presented in the article written by Schiffmann et al^11^ These disorders where afterward excluded using clinical and biological findings.

1. CADASIL (Cerebral Autosomal Dominant Arteriopathy With Subcortical Infarcts and Leukoencephalopathy) usually has an earlier onset, during the third decade. The lack of findings in the family history of our patient, the absence of lesions on imaging anterior to the temporal horns of the lateral ventricles and of the migraines makes this diagnosis improbable. Also, the presence of vascular risk factors (arterial hypertension and smoking) plead for an acquired microangiopathy.^9^, ^11^
2. Multifocal progressive leukoencephalopathy may present itself with similar imaging features to Binswanger's Disease, but cerebral lacunes should be absent 9. Characteristic lesions on MRI are more often localized at the gray‐white matter junction with damage to the juxtacortical association fibers.^12^ On clinical examination, motor coordination and gait disturbances are frequently found alongside visual field deficits and language abnormalities.^12^ A normal hematological and immunological profile and the absence of opportunistic infections and other infectious events in the past that would suggest an acquired or intrinsic immunodeficiency are also favorable for excluding this diagnosis.^13^
3. Cerebral vasculitis as part of a systemic disorder may have various features on MRI imaging, and the clinical and biological features of the disease include: signs of a systemic disease, headaches, elevated acute‐phase reactants, inflammatory microcytic anemia, and the presence of autoantibodies.^14^ None of these changes were found in our patient. Antinuclear antibodies, antineutrophil cytoplasmic antibodies, rheumatoid factor, and circulating immune complexes were all in normal range.
4. Primary angiitis of the central nervous system (PACNS) is rare disorder and should be suspected in younger patients with strokes, without any known vascular risk factors. The major symptoms of this disease include headaches (60% of cases), cognitive impairment (50% of cases), and neurological focal signs. Systemic and biological changes that were described in the cases of systemic vasculitis are often absent. On MRI, the lesions are typically multifocal, bilateral, hyperintense on T2WI and FLAIR sequences, and can be found in the superficial and deep white matter, and in the cortical gray matter and basal ganglia.^15^ Of cardinal importance are the bilateral stenosis and dilation of various sized blood vessels^15^ which were absent in our patient. The gold standard for diagnosing or excluding PACNS is represented by cerebral biopsy,^15^ which we preferred not to perform due to lack of sufficient arguments that would justify the risk of the investigation.
5. Toxic leukoencephalopathy develops in the context of chronic exposure to leukotoxic substances such as some illicit drugs (opioids, cocaine, amphetamines). Leukoencephalopathy caused by use of heroin, also known as "chasing the dragon" syndrome, is one of the most studied of these rare occurrences, and it usually presents on MRI with white matter hyperintensities that are symmetrical, especially with changes in the corticospinal tracts of the pons, perirolandic white matter, cerebellar white matter with sparing of the juxtacortical white matter and the gray matter.^16^, ^17^ These lesions are similar to those seen in our case. The patient denied any use of illicit substances, did not have any signs of chronic toxicity of the substances mentioned above, and did not develop any withdrawal events during the 2‐week hospitalization period. Toxic leukoencephalopathy may also develop after chronic exposure to toluene. Our patient was a painter and toluene can be found in many paints and solvents. The main neurological feature of chronic toluene toxicity is the cognitive impairment.^18^ The patient refused to give any information about the ingredients of the paints used by him. Nevertheless, the presence of other lesions associated on MRI, other than the white matter hyperintensities, are not typical in chronic toluene toxicity. There were also no other systemic disturbances that would suggest chronic toluene toxicity (altered kidney function, respiratory, dermatological, gastrointestinal, or musculoskeletal abnormalities).
6. HIV‐associated dementia is associated with a symmetrical, periventricular pattern of changes to the white matter with sparing of the juxtacortical and infratentorial white matter, atrophy of the gray matter of the cerebral cortex, atrophy of the deep white matter and volumetric changes of the basal ganglia,^19^ and changes that were not present in our patient. Also, the lack of criteria that would suggest and acquired immunodeficiency as mentioned previously makes this diagnostic much less probable.
7. Other illnesses that have a genetic etiology are usually detected during childhood or adolescence and are frequently associated with systemic abnormalities. Out of these pathologies, Fabry disease is still worth mentioning. There are some rare cases described in literature, in men, that had a late‐onset at adult age and in which only one or two organ systems were affected. The kidneys (chronic kidney disease) and the cardiovascular system (hypertrophic cardiomyopathy, valvular anomalies, conduction disorders) were most frequently affected.^20^, ^21^ Our patient had no clinical or paraclinical evidence of a kidney or heart abnormalities. When present, central nervous system affliction leads to frequent ischemic strokes and cognitive impairment. On MRI, white matter lesions are present in 80% of cases and can be heterogenous, ranging from small, scattered, and punctuate T2‐weighted hyperintense foci to bilateral diffuse, patchy, and partly confluent white matter hyperintensities. An important characteristic is that the lesions in Fabry disease only present themselves in the supratentorial white matter, which was not the case in our patient.^20^

### 2.2 Outcome and follow‐up

The therapeutic management targeted lowering of blood pressure in accordance with the actual guidelines and secondary stroke prevention using antiplatelet monotherapy and statins. Blood pressure was especially difficult to control requiring multiple associations of anti‐hypertensive drugs. Rosuvastatin 20 mg per day and clopidogrel 75 mg per day were started in the first 24 hours. Urapidil iv was used to control the blood pressure during the first 48 hours. We started with 3 doses of 12.5 mg each at 10‐minute intervals followed by continuous infusion at a variable rate (10‐30 mg per hour) in order to slowly reduce and maintain the blood pressure at 180/110 in the first 24 hours. Indapamide 1.5 mg per day, Perindopril 5 mg per day, and Amlodipine 5 mg per day were also added, and the dosages were adjusted in order to maintain the blood pressure at 130‐140/80‐90 mm Hg during the following weeks. The patient was also evaluated in the first 72 hours after admission by a kinetotherapist, and daily sessions of active and passive mobilization were performed. The patient was referred to a cardiologist after discharge.

Regarding the clinical and neurological evolution, the motor deficit, fine motor control, and prehension improved during admission and during the first month after discharge from hospital.

## 3 DISCUSSION

A diagnosis of Binswanger's disease was proposed for our patient based on the clinical features regarding the risk factors, general and neurological examination, the white matter changes observed on CT and MRI, and also a thorough differential diagnosis. More than 20 years have passed since Benett and Caplan proposed a diagnostic criterion for Binswanger's disease.^2^ The pathophysiology of the disease has been better understood since then and besides the usual clinical features and imaging, ancillary tests can be used in difficult cases.^5^

Changes in CSF biochemistry may reflect the neuroinflammation present in small vessel disease. Neuroinflammation leads to blood‐brain barrier dysfunction with increased permeability on the one hand and important changes in the protein and cytokine expression patterns in glial cells on the other hand. These changes may be reflected by an increased albumin index in the CSF (due to increased permeability) and in increased levels of inducible matrix metalloproteinases, such as MMP‐3 and MMP‐9 (due modified protein expression).^5^, ^6^, ^22^

A recent biomarker identified as having larger levels is patients with Binswanger's disease is lipocalin 2 (LCN2). LCN2, also known as oncogene 24p3, a glycoprotein involved in NVU damage in patients with vascular disease. It had promising results and was found having larger levels in patients with vascular dementia as opposed to Alzheimer's disease or other types of dementia.^23^

Various imaging studies such as MRI diffusion tensor imaging (to evaluate white matter tracts integrity) or dynamic contrast enhancement MRI (to reveal disruption of the blood‐brain barrier) can aid the clinician in establishing the diagnosis. These imaging techniques and many others have unknown reproducibility and lack validation for Binswanger's disease in larger populations. It is important to mention that the diagnosis cannot be given solely on CT or MRI imaging and requires a careful clinical examination.^5^, ^22^, ^24^

Establishing the diagnosis for Binswanger's disease requires a multimodal approach. None of the biomarkers alone are adequate to diagnose the disease, but using clinical data alongside imaging and ancillary tests can prove helpful in patients with cognitive impairment and neurological signs with uncertain or unknown etiology.^5^, ^6^, ^22^, ^24^

The literature is scarce regarding the prognosis of the disease. Binswanger's disease is progressive, and there is currently no cure available. There are no specific clinical studies targeting therapies for Binswanger's disease, although the American heart association (AHA) published treatment guidelines for patients with vascular cognitive impairment.^25^ blood pressure control, antiplatelet therapy, and statins play a central role in secondary stroke prevention and lead to slowing the progression of white matter lesions. Dietary changes (especially reducing the intake of salt), physical activity, physical therapy, and rehabilitations are an important part in the management of these individuals, leading to improvement of the functional status and the quality of life, besides other well known health benefits.^5^, ^26^, ^27^

## 4 CONCLUSIONS

Binswanger's disease is a complex neuropsychiatric disease, and its pathophysiology is only partially understood. As new pathophysiological mechanisms are revealed, other tests will become available in the years to come and also novel therapies will specifically target these mechanisms (inflammation, arterial stiffness, and clearance of cerebral waste) in order to better treat these patients.

The particularity of our case resides in the fact that the white matter lesions were diffuse, extending from infratentorial to the brainstem, cerebellar peduncles and the cerebellum, which is not common in Binswanger's disease and is rarely described in literature.

## CONFLICT OF INTEREST

The authors declare that there was no conflict of interest regarding the publication of this paper.

## AUTHOR CONTRIBUTIONS

VV: head of department, consultant neurologist in charge of the patient. Given approval of the final version. AMC: resident doctor, conception and design of the case report. IR: resident doctor, revising the case report and drafting the discussion section. SF: resident doctor, revising the case report and comparing it to the current literature. DFM: coordinator of the team.

## ETHICAL APPROVAL

Ethics committee approval and patient informed consent were obtained prior to submitting the manuscript.
